# Low Gestational Weight Gain Skews Human Sex Ratios towards Females

**DOI:** 10.1371/journal.pone.0114304

**Published:** 2014-12-10

**Authors:** Kristen J. Navara

**Affiliations:** Department of Poultry Science, The University of Georgia, Athens, Georgia, United States of America; University Medical Center Utrecht, Netherlands

## Abstract

**Background:**

Human males are more vulnerable to adverse conditions than females starting early in gestation and continuing throughout life, and previous studies show that severe food restriction can influence the sex ratios of human births. It remains unclear, however, whether subtle differences in caloric intake during gestation alter survival of fetuses in a sex-specific way. I hypothesized that the ratio of male to female babies born should vary with the amount of weight gained during gestation. I predicted that women who gain low amounts of weight during gestation should produce significantly more females, and that, if gestational weight gain directly influences sex ratios, fetal losses would be more likely to be male when women gain inadequate amounts of weight during pregnancy.

**Methods:**

I analyzed data collected from over 68 million births over 23 years to test for a relationship between gestational weight gain and natal sex ratios, as well as between gestational weight gain and sex ratios of fetal deaths at five gestational ages.

**Results:**

Gestational weight gain and the proportion of male births were positively correlated; a lower proportion of males was produced by women who gained less weight and this strong pattern was exhibited in four human races. Further, sex ratios of fetal losses at 6 months of gestation were significantly male-biased when mothers had gained low amounts of weight during pregnancy, suggesting that low caloric intake during early fetal development can stimulate the loss of male fetuses.

**Conclusion:**

My data indicate that human sex ratios change in response to resource availability via sex-specific fetal loss, and that a pivotal time for influences on male survival is early in fetal development, at 6 months of gestation.

## Introduction

It has been well-demonstrated that, at all gestational ages, male fetuses are more susceptible to adverse conditions in the womb than females [1]. Males have an overall higher risk of miscarriage and pre-term birth [2]–[4]. In addition, male and female embryos are different metabolically and grow at different rates [6]–[8]. While recommendations regarding calorie and nutrient intakes for pregnant women are currently universal for women carrying both males and females, it is important to examine whether different levels of nutrient and calorie-intakes affect the sexes differently.

Male embryos start life with higher rates of cell division and higher metabolic rates compared to females [5], [6]. These differences appear to extend through the second trimester, as male fetuses exhibit faster growth rates prior to the third trimester, after which the sex differences in growth rates appear to even out [7]. The result is a nearly 100 g weight difference between male and female newborns [8]. This disparity in growth rates between males and females indicates that nutritional requirements should also be sex-specific. Indeed, women carrying boys had 10% higher energy intake compared to those carrying girls [8]. Women with diseases that lower food intake or nutritional absorption such as anorexia, bulimia, and celiac disease, produce lower percentages of male offspring [9], [10]. Further, during times of famine, the sex ratio at birth declines, becoming female-biased [11], [12], Ethiopian women that were in a better nutritional state according to body mass and muscle indices had a higher percentage of male births, and Italian women who were thinner produced a lower percentage of boys [13]. On the other hand, women with binge-eating disorders produced significantly higher percentages of males [9] and rich individuals who likely have access to larger amounts of nutritious food also produce more male babies [14], [15]. The calorie intake per capita is also related to sex ratios at birth; countries with lower caloric intakes produce lower percentages of males at birth, likely due to higher rates of male fetal death [16]. These studies suggest that extreme decreases in energy intake are detrimental to the survival of male fetuses.

Studies in non-human mammals support the idea that fewer males are produced when food intake is suboptimal. For example, dairy cows on a highly nutritious diet produced a higher proportion of bulls than those on a poor diet [17]. Similarly, mice fed a diet very high in fat produced a high proportion of males (0.67) while mice fed a low fat diet produced a low proportion of males (0.39) [18]. To date, however, few studies have tested for a relationship between subtle differences in energy intake, weight gain, and birth sex ratios in humans. In one study, women carrying males had higher caloric intakes during gestation but there was no relationship between gestational weight gain and embryo sex [8]. In contrast, another study showed that women carrying *female* babies had higher intakes of energy and saturated fat during the first trimester [19]. Thus, it remains unclear whether subtle differences in food intake and weight gain during gestation differentially influence male survival *in utero* and, if so, when during gestation these influences would be most potent.

Previous studies indicate that sex-specific fetal death is the most common mechanism by which human sex ratios are skewed [20]. It is possible that control of sex ratios could lie within the male via differential production of X or Y-bearing sperm [21], [22], or by altering the quality of sperm carrying either and X or Y chromosome such that one is more likely to fertilize than the other [22]. Alternatively, control may lie within the female, for example if the uterine environment is more hostile to X or Y-bearing sperm [22], if the oocyte is more susceptible to X or Y-bearing sperm at fertilization, or if male and female blastocyst have different mortality rates prior to or after implantation [18], [23]. Indeed, in one human study, pre-conceptual diet was significantly related to the sex ratio at birth [24]. However Young et al. [25] suggest that this pattern results from multiple testing, and work on non-human mammals by Rosenfeld [26] suggests that diet does not alter sex ratios via changes in X versus Y bearing sperm, differential fertilization, or sex-specific mortality in the pre-implantation period. Instead, a majority of studies on humans showing effects of adverse conditions on sex ratios suggest sex-specific fetal deaths as the mechanism (reviewed in [20]), and this mechanism is the focus of the current study.

I hypothesized that the amount of calories taken in during gestation, as indicated by gestational weight gain, would predict the survival of male and female offspring and, ultimately, the ratio of male versus female offspring born. I predicted that gestational weight gain would be positively related to the percentage of male babies born. Further, if the relationship between natal sex ratios and gestational weight gain result from sex-specific fetal death within these weight categories, I would expect to see the opposite relationship between gestational weight gain and the sex ratio of fetal deaths; fetuses lost to women who gained inadequate amounts of weight would be more likely to be male, and thus sex ratios in this group should be higher.

## Materials and Methods

All data described herein were collected from the Center for Disease Control and Prevention Vital Statistics website (CDC 2014) [27], and I utilized data for all years available for all analyses. To test whether natal sex ratios were significantly related to gestational weight gain, I collected data on >68 million singleton births in the US from 1990–2012, as well as the gestational weight gain for each pregnancy in pounds, the sex of the infant, the gestational age of the infant at the time of birth, and the race of the mother. Only infants that were considered full-term (gestational age ≥37 weeks) were included in the analyses. It has been shown repeatedly that gestational weight gain is a good indicator of total caloric energy intake during gestation [28]–[32]. Gestational weight gain was available in increments of 1 lb up to 97 pounds; all gains below or above 98 lbs were excluded because they were lumped into categories of “below 1 lb” or “above 98 lbs”, and specific weight gains were unclear. To analyze the relationship between gestational weight gain and the proportion of male offspring produced, I conducted a binary logistic regression analysis. In addition, I ran the same analyses separately for individuals in each of four races provided by the CDC, including women of American Indian, Asian, Black, and White descent. This is because natal sex ratios vary significantly between races (American Indian: 50.2%, Asian: 50.9%, Black: 50.5%, White: 50.5% male, F_3,87_  = 102.68, p<0.0001).

To test whether sex ratios of fetal deaths differed based on the level of gestational weight gain, I collected total fetal deaths in the US from 2003–20012, as well as the sexes of these fetuses and the gestational weight gains associated with each. Because gestational weight gains differed based on when in gestation fetal deaths occurred, and to determine whether the sex ratios of fetal deaths were related to gestational weight gains at particular gestational stages, I divided fetal death data into the following available months of gestation: 6 months (weeks 21–24), 7 months (weeks 25–28), 8 months (weeks 29–32), 9 months (weeks 33–36), and 10 months (weeks 37–40). I analyzed the relationship between gestational weight gain and fetal sex during each gestational month using binary logistic regression analyses.

Finally, to test whether natal sex ratios were significantly related to pre-pregnancy Body Mass Index (BMI), I collected sex ratio and BMI data for all US women from 2011–2012. Women were grouped into three BMI categories according to BMI classifications published by the World Health Organization (WHO) [33]: low (<18.4), average (18.5–24.9) and high (>25). I calculated the average natal sex ratio for each category using data from 1990–2012 and then ran individual Fishers Exact Tests to test for differences in natal sex ratios among BMI groups. All analyses were run using SAS 9.0.2 (SAS Institute) and relationships with alphas at or below 0.05 were considered significant.

## Results

**Figure 1:**
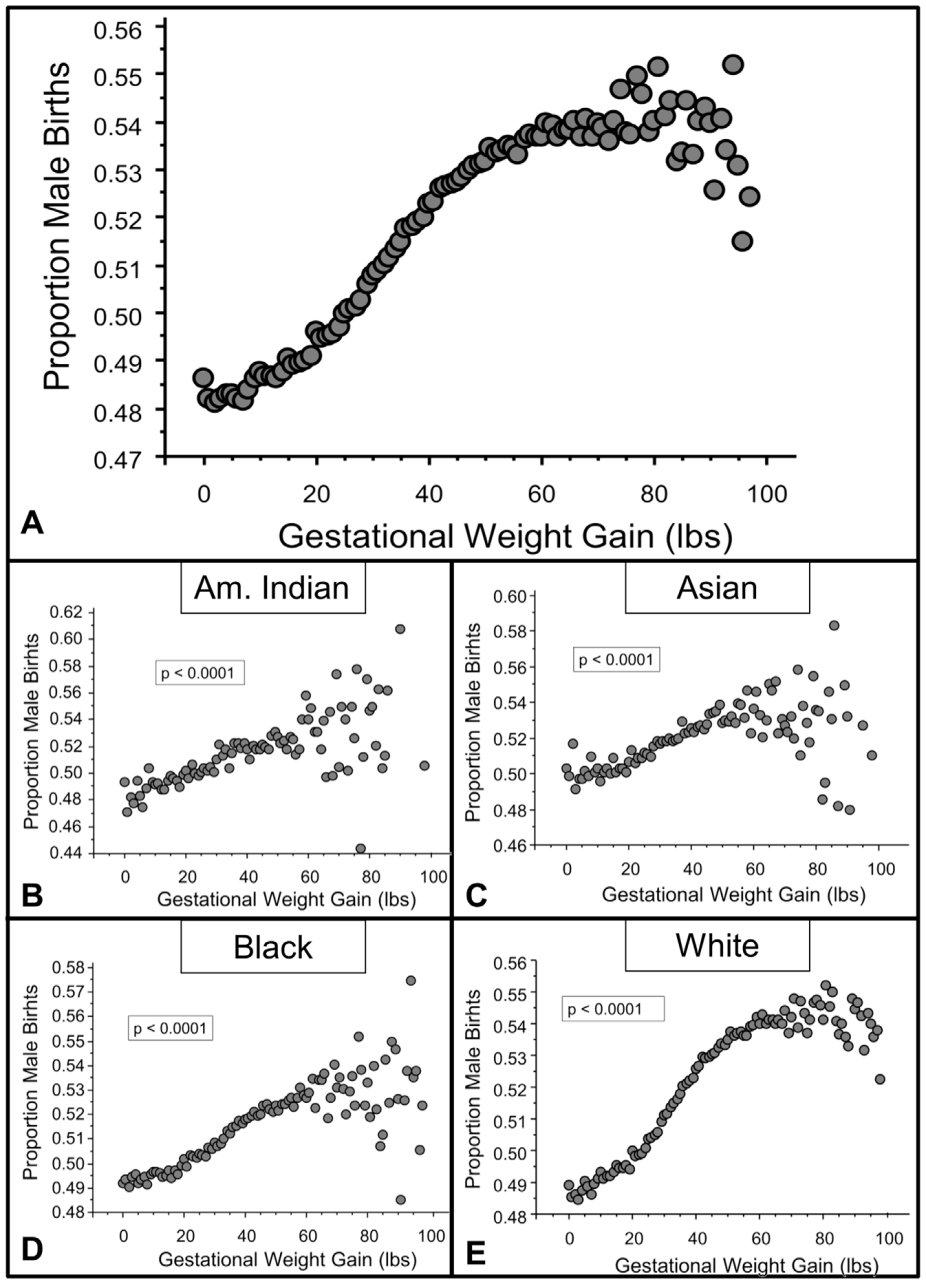
The relationship between gestational weight gain and the proportion of male births. (A) This plot shows the proportion of male births for women of all races in relation to gestational weight gain. Each data point represents the average proportion of male births for a given weight gain calculated using data collected by the Center for Disease Control from 1990–2012. Also included are individual plots for individuals of American Indian (B), Asian (C), Black (D), and White (E) descent. Alpha values were derived from binary logistic regression analyses.

There was a significant positive correlation between gestational weight gain and the natal sex ratio (Wald's χ^2^ = 45,254.00, p<0.0001, Fig. 1A). At about 60 lbs of weight gain, the fit of the model decreased, and when only gains greater than 60 lbs were analyzed in relation to the natal sex ratio, there was no relationship (Wald's χ^2^ = 1.82, p = 0.18). Thus gestational weight gain and natal sex ratio are significantly correlated, but once weight gains reach 60 lbs, there is no influence of additional weight gains on natal sex ratio. Women who gained more weight during gestation produced a significantly higher proportion of male babies. The positive relationship was similar for women in all four race categories provided by the CDC (American Indian: Wald's χ^2^ = 457.54, p<0.0001, Asian: Wald's χ^2^ = 1034.87, p<0.0001, Black: Wald's χ^2^ = 4642.38, p<0.0001, White: Wald's χ^2^ = 45,5 = 254.00, p<0.0001 Fig. 1B, C, D, and E). For White and Asian births, the fit of the model changed at 60 lbs, just as it did when all births were included in the analysis; in these two rates, there was no additional change in sex ratios with weight gains beyond 60 lbs.

**Figure 2:**
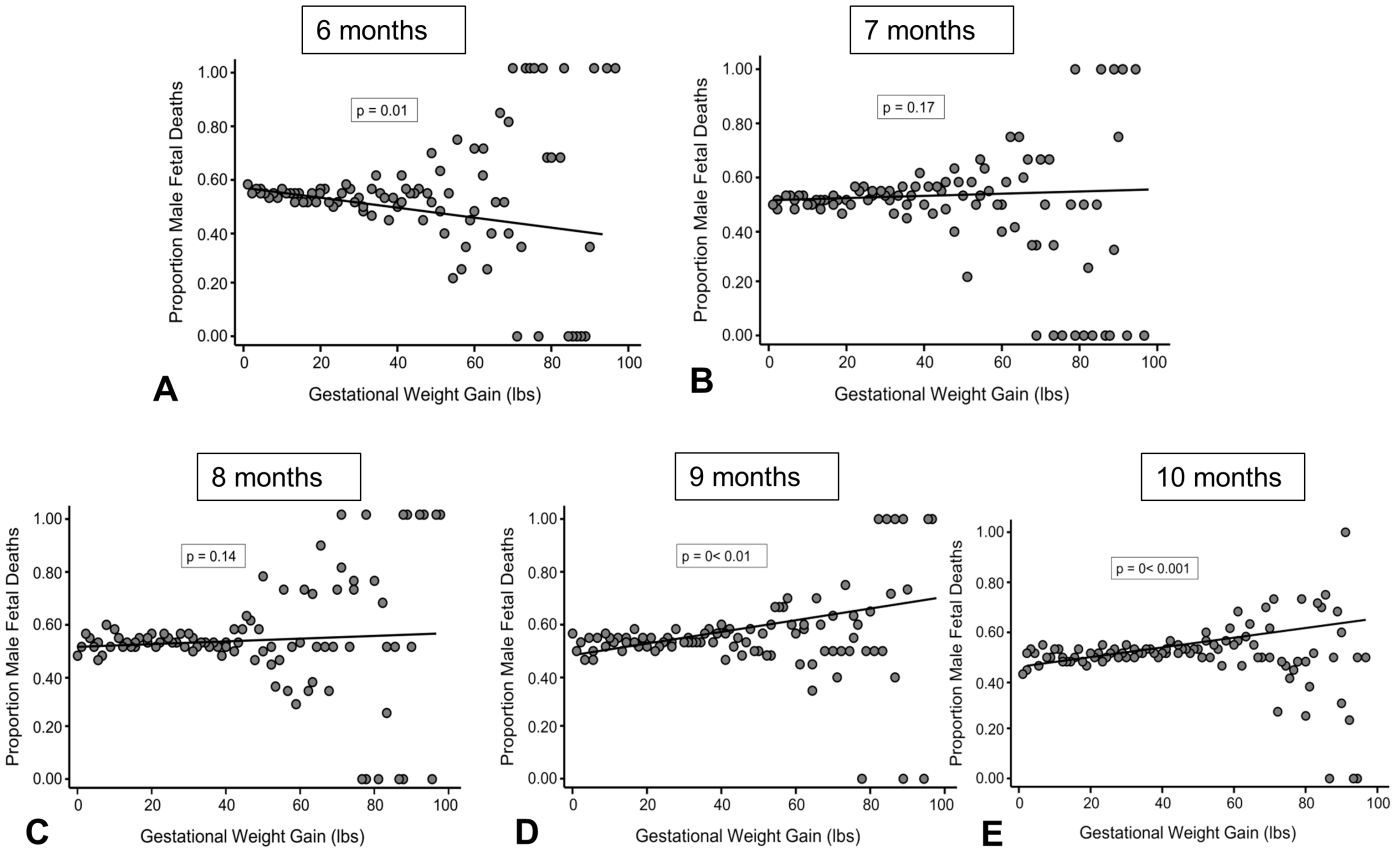
Relationships between gestational weight gains and sex ratios of fetal deaths. (A) Shows this relationship at 6 months (weeks 21–24), (B) at 7 months, (C) at 8 months, (D) at 9 months, and (E) at 10 months of gestational age. Each data point represents the average proportion of male births for a given weight gain calculated using data collected by the Center for Disease Control from 2003–2012. Alpha values were derived from binary logistic regression analyses.

The relationship between gestational weight gain and the sex ratios of fetal deaths at 6 months of gestation was the opposite of that seen with births; gestational weight gain was negatively correlated with the sex ratios of fetuses at this gestational age (Wald's χ^2 = ^6.38, p = 0.01, Fig. 2A). At 7 and 8 months of fetal age, there was no relationship between gestational weight gain and the sexes of recovered fetuses (7 months: Wald's χ^2^ = 1.80, p = 0.17, 8 months: Wald's χ^2^ = 2.12, p = 0.14, Fig. 2B & C). By 9 and 10 months of age, sex ratios of recovered fetuses were significantly positively correlated to gestational weight gains, similar to the relationship seen with natal sex ratios (9 months: Wald's χ^2^ = 7.60, p = 0.006, 10 months: Wald's χ^2^ = 17.79, p<0.0001, Fig. 2D & E).

**Figure 3:**
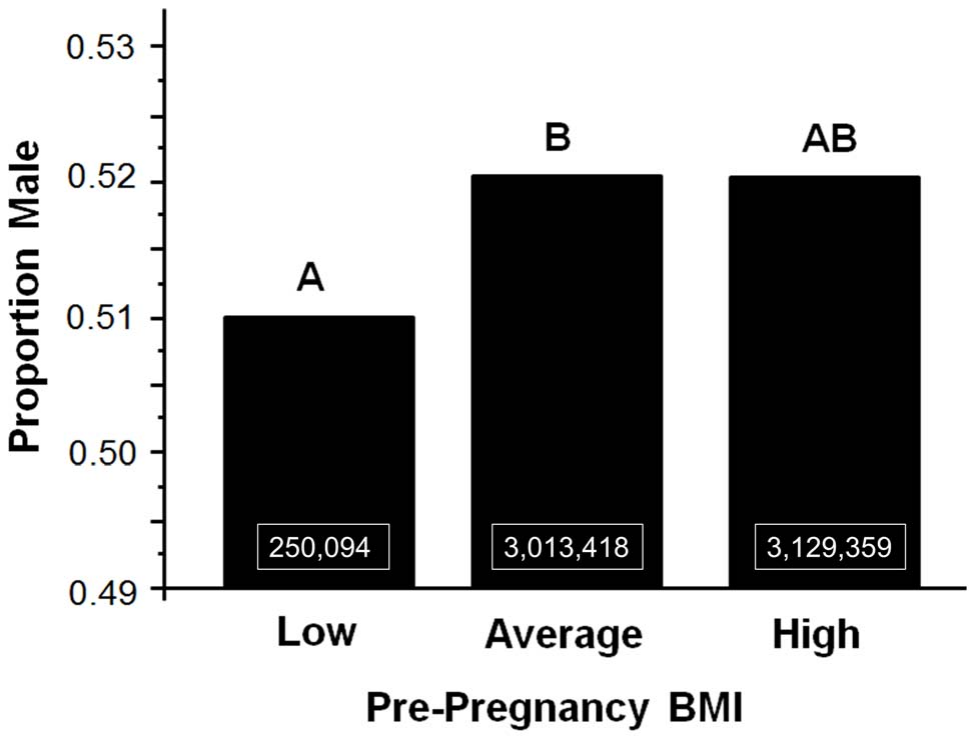
Relationship between full-term natal sex ratios and pre-pregnancy BMI. Different capital letters indicate significant differences.

Women with a low BMI (<18.4) pre-pregnancy produced significantly fewer males than those with an average BMI (18.4–24.9)(χ^2^ = 4.94, p = 0.03), however there was no difference between sex ratios produced by women with low and high BMIs (Fig. 3)(χ^2^ = 2.84, p = 0.09). When sex ratios produced by women with low pre-pregnancy BMIs were compared with those from all other women, women with low pre-pregnancy BMIs produced significantly fewer males (χ^2^ = 31682, p<0.0001).

## Discussion

**Figure 4:**
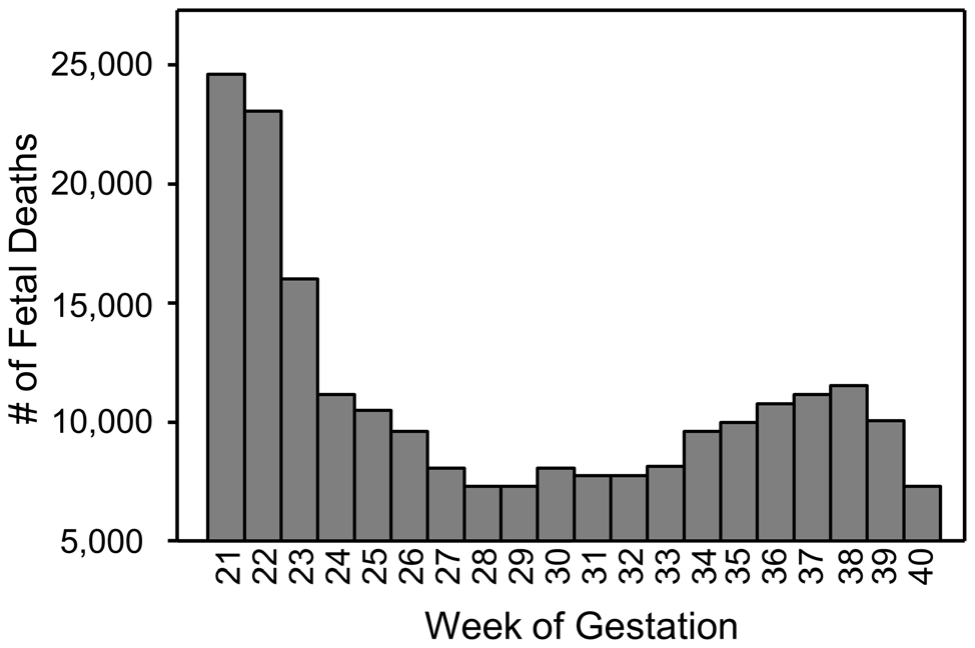
Numbers of fetal deaths that occurred at each week of gestation.

As predicted, women who gained low amounts of weight during gestation produced a significantly lower proportion of male offspring compared with those that gained high amounts of weight, and this effect was similar for women in all four races studied. It is possible that this relationship results from the fact that male embryos and fetuses have higher metabolic rates, and likely need more caloric energy to develop successfully [6]–[8]. Under this scenario, because the metabolic differences between the sexes are evident very early in development [6]–[8], we would expect to see a similar positive relationship between gestational weight gain and the proportion of male fetuses at any gestational age, particularly if the relationship between weight gain and fetal sex resulted from an inherent difference in the way women carrying male offspring gain weight. However, at 6 months of gestational age, a time when the number of fetal deaths was particularly high (Fig. 4), there was instead a significant *negative* correlation between gestational weight gain and the proportion of male fetuses recovered, suggesting that gestational weight gain may be influencing survival of fetuses in a sex-specific way, and that low weight gains may be more detrimental to male fetuses. There was no relationship between gestational weight gain and the proportion of male fetal deaths at 7 and 8 months of gestation, and even this lack of a relationship may be important, because if gestational weight gain did not have an influence on fetal death in a sex-specific manner, we would have expected a sampling of fetuses at any gestational age would show the same positive relationship between weight gain and sex ratios that we saw in the birth data. It wasn't until 9 and 10 months of gestational age that relationship reflected that of the birth sex ratio; gestational weight gain was positively related to the proportion of male fetal deaths at this late stage, just as it was for the babies born alive at similar gestational ages. The CDC only provided data for fetal deaths from 6 months to 10 months of gestation, but it appears likely that the negative relationship seen between weight gains and fetal sex ratios at 6 months would extend even earlier in gestation. Future studies will need to address this.

Also of interest is the fact that the analyses of Asian, White, and all combined births shows a change in the model fit above 60 lbs of gestational weight gain. Above 60 lbs, the proportion of male offspring no longer changes as weight gain increases. It could be that there is a ceiling to the benefit of weight gain for developing male fetuses, and that once optimal weight gain has been reached, there is no additional benefit to male fetuses. It is also possible that weight gains at the very high end of the spectrum are in fact detrimental to male fetuses; indeed the proportion of males produced appears to drop slightly towards the higher end of the weight gain range (Fig. 1A). However, when I examined the relationship between weight gain and the proportion of male births for weight gains above 60 lbs, there was no significant relationship. I would have expected to see a negative relationship if higher weight gains were, in fact, detrimental to males.

It has been well-documented that the average natal sex ratios for most human populations studied are slightly male-biased, while the ratios of X: Y bearing sperm do not appear to differ from 50∶50 [34]. The male-bias in the natal sex ratio is likely reached through two steps: first, female blastocysts appear to be particularly fragile prior to implantation, during the initial stages of embryogenesis, as sex ratios of embryos spontaneously aborted at the earliest stage of development are highly female-biased (31% male) [35]. As a result, after implantation, sex ratios of living conceptuses are likely to be highly male-biased, however male embryos are especially vulnerable to adverse conditions *in utero* throughout the remainder of gestation [35]–[37], which lowers the final natal sex ratio to approximately 51.2% males. Indeed in my study, the natal sex ratio remained between 51.1% and 51.2% for all 23 years studied, yet sex ratios of fetal deaths were even more male-biased (52.6% male). The highest percentage of males was found in fetuses recovered earlier than 7 months (54.0% male), which suggests that male embryos are particularly vulnerable at this early fetal stage. Thus it is not surprising that an inadequate weight gain during this time period would result in an even higher proportion of male fetal deaths, and ultimately a lower proportion of live-born males.

Using these data, we cannot definitively determine that low gestational weight gain was directly responsible for changes in sex ratios of births and fetal deaths. Previous studies have shown that energy intake [24] and weight [13], [38] prior to conception can may influence offspring sex ratios, and women with high body mass index (BMI) prior to pregnancy are more likely to gain more during gestation as well [39], [40]. We tested whether pre-pregnancy BMI was significantly related to natal sex ratios. Women with a low BMI (<18.4) pre-pregnancy produced significantly fewer males than those with an average BMI (18.4–24.9), however there was no difference between sex ratios produced by women with low and high BMIs (Fig. 3). These patterns did not likely drive the relationship between gestational weight gain and natal sex ratios, because natal sex ratios in the high BMI group were only 0.2% higher than in the remaining two groups while differences between high and low gestational weight gain groups were 2–4%, a much stronger effect. Instead, it is likely that the relationship between natal sex ratios and pre-pregnancy BMI were driven by the stronger relationship between natal sex ratios and gestational weight gain. It is still possible, however, that another factor related to gestational weight gain is ultimately responsible for the skews in sex ratios, however given the strength of the relationship described here, I suggest that it is unlikely that the relationship between gestational weight gain and natal sex ratios is an indirect effect.

I also cannot rule out the possibility that the relationship between gestational weight gain and sex ratio is driven by the fact that women gain more weight when carrying a male fetus. Indeed, in this study, women gained on average 0.5 lbs more when the fetus was male. Further, Caulfield et al. [41] showed that women carrying male fetuses were more likely to over-gain. However, if the relationships were driven by the higher weight gains of male babies, I would have expected the sex ratio data points to fall into two relatively distinct weight gain ranges, rather than the slow progression I saw here, where natal sex ratios increased with every pound of weight gain during gestation. I suggest that it is unlikely that subtle differences in food intake between women carrying boys and girls would explain the relationship seen here. In addition, the reverse relationship where sex ratios of fetal deaths at 6 months weeks were inversely related to gestational weight gains would not be expected if the relationships are driven by differential weight gain between women carrying boys and girls. Instead I would expect to see the opposite pattern.

It appears that human females that take in fewer calories during gestation are adjusting sex ratios via sex-specific fetal death, and this complies with previous work in humans showing that severe food restrictions decrease the proportion of male babies born [9]–[12]. It is still unclear, however, whether this represents an adaptive strategy of sex allocation. Trivers and Willard [42] theorized that mothers in the best condition should produce offspring with the highest variance in reproductive success (RS), while those in poor condition should produce more of the sex with low variance in RS. While applying this concept to humans is complicated due to the high level of parental investment provided by males and the relatively monogamous nature of humans, several studies (reviewed in [18]) support that idea that humans employ the adaptive strategy of sex ratio allocation posited by Trivers and Willard [42]. Indeed, in the current study, women who gained large amounts of weight during gestation produced a higher proportion of male offspring. For the sex ratio adjustments produced by humans to be adaptive in nature, the skews must be biologically significant. The percentages of male births in this study ranged from 48–55% across the range of gestational weight gains. This appears to be a relatively narrow range, however other studies in mammals indicating the presence of sex allocation show similar ranges (Ungulates: [43], [44], Primates: [45], [46]). In addition, the range I show here is an order of magnitude greater than those shown in many human sex ratio studies (e.g. [13], [47], [48], but see [14], [24]). It is also possible, however, that this pattern is not adaptive in nature, and simply occurs as a side effect of the higher metabolic demands associated with male gestation. More studies are needed to clarify the adaptive significance, if any, of the pattern seen here. In addition, more work needs to be done to examine how lower intakes of particular dietary components may influence survival in a sex-specific way.
